# Development of ensemble learning models for prognosis of hepatocellular carcinoma patients underwent postoperative adjuvant transarterial chemoembolization

**DOI:** 10.3389/fonc.2023.1169102

**Published:** 2023-05-26

**Authors:** Yuxin Liang, Zirui Wang, Yujiao Peng, Zonglin Dai, Chunyou Lai, Yuqin Qiu, Yutong Yao, Ying Shi, Jin Shang, Xiaolun Huang

**Affiliations:** ^1^ Liver Transplantation Center and Hepatobiliary and Pancreatic Surgery, Sichuan Cancer Hospital and Institute, Sichuan Cancer Center, School of Medicine, University of Electronic Science and Technology of China, Chengdu, China; ^2^ Department of Hepatobiliary-Pancreatic Surgery, Cell Transplantation Center, Sichuan Provincial People’s Hospital, University of Electronic Science and Technology of China, Chengdu, China; ^3^ School of Computer Science and Engineering, University of Electronic Science and Technology of China, Chengdu, China

**Keywords:** machine learning, hepatocellular carcinoma, postoperative adjuvant TACE, recurrence, prognosis

## Abstract

**Background:**

Postoperative adjuvant transarterial chemoembolization (PA-TACE) has been increasing widely used to improve the prognosis of hepatocellular carcinoma (HCC) patients. However, clinical outcomes vary from patient to patient, which calls for individualized prognostic prediction and early management.

**Methods:**

A total of 274 HCC patients who underwent PA-TACE were enrolled in this study. The prediction performance of five machine learning models was compared and the prognostic variables of postoperative outcomes were identified.

**Results:**

Compared with other machine learning models, the risk prediction model based on ensemble learning strategies, including Boosting, Bagging, and Stacking algorithms, presented better prediction performance for overall mortality and HCC recurrence. Moreover, the results showed that the Stacking algorithm had relatively low time consumption, good discriminative ability, and the best prediction performance. In addition, according to time-dependent ROC analysis, the ensemble learning strategies were found to perform well in predicting both OS and RFS for the patients. Our study also found that BCLC Stage, hsCRP/ALB and frequency of PA-TACE were relatively important variables in both overall mortality and recurrence, while MVI contributed more to the recurrence of the patients.

**Conclusion:**

Among the five machine learning models, the ensemble learning strategies, especially the Stacking algorithm, could better predict the prognosis of HCC patients following PA-TACE. Machine learning models could also help clinicians identify the important prognostic factors that are clinically useful in individualized patient monitoring and management.

## 1 Introduction

Liver cancer is the sixth most prevalent malignancy and the third leading cause of cancer-related death worldwide (1, 2). By 2025, the estimated incidence of liver cancer may exceed 1 million (3). Hepatocellular carcinoma (HCC) is the most common primary liver cancer, accounting for about 75%-85% (4). Although curative hepatectomy is still recommended as the main curative treatment for HCC patients with adequate liver function (5, 6), postoperative prognosis of HCC patients is jeopardized by a high recurrence rate (7). Therefore, several postoperative adjuvant therapies have been developed to reduce the risk of recurrence and improve overall survival (8–10). Transarterial chemoembolization (TACE), which has long been one of the first-line treatments for unresectable HCC (11), is now most widely used as an adjuvant therapy after curative resection for HCC with many recurrence risk factors (12–14). Substantial studies have also shown that postoperative adjuvant TACE (PA-TACE) is beneficial for HCC patients with more tumor numbers, larger tumor size, and microvascular invasion (15–18), especially for those with portal vein tumor thrombus (19). However, few studies have established effective and practical prediction models for prognosis of HCC patients underwent PA-TACE and achieved satisfactory prediction efficacy. Therefore, novel prediction models are needed to facilitate clinical decision making in early management and further improve patient outcomes.

Machine learning, a type of computer science that makes empirical predictions from multi-dimensional datasets, is increasingly being applied to modern medical research, including HCC (20–23). In the prognostic prediction of HCC, it has shown superior advantages in image recognition and feature selection compared with traditional methods, thereby improving the accuracy of the model prediction and subsequent results (21, 22, 24). The rise of artificial intelligence (AI) technology has also brought many new machine learning strategies to predict patient prognosis. In recent researches, several ensemble learning strategies, including Boosting and Bagging algorithm have been developed for HCC and achieved encouraging results (25–27). Different from other machine learning methods that typically apply one model or one algorithm to a specific task, ensemble learning performs greater flexibility in model selection. Specific training strategies could be set for complex clinical datasets to improve the performance of the ensemble learning strategy.

In summary, this study aimed to utilize and compare different machine learning algorithms to establish a better prediction model for survival and recurrence of HCC patients underwent PA-TACE. Five machine learning models, including three novel ensemble learning models and two other models, were selected to provide intelligent postoperative monitoring and management for the patients. We also explored the variable importance and verified important prognostic indicators of postoperative outcomes.

## 2 Materials and methods

### 2.1 Patients and study design

**Figure 1:**
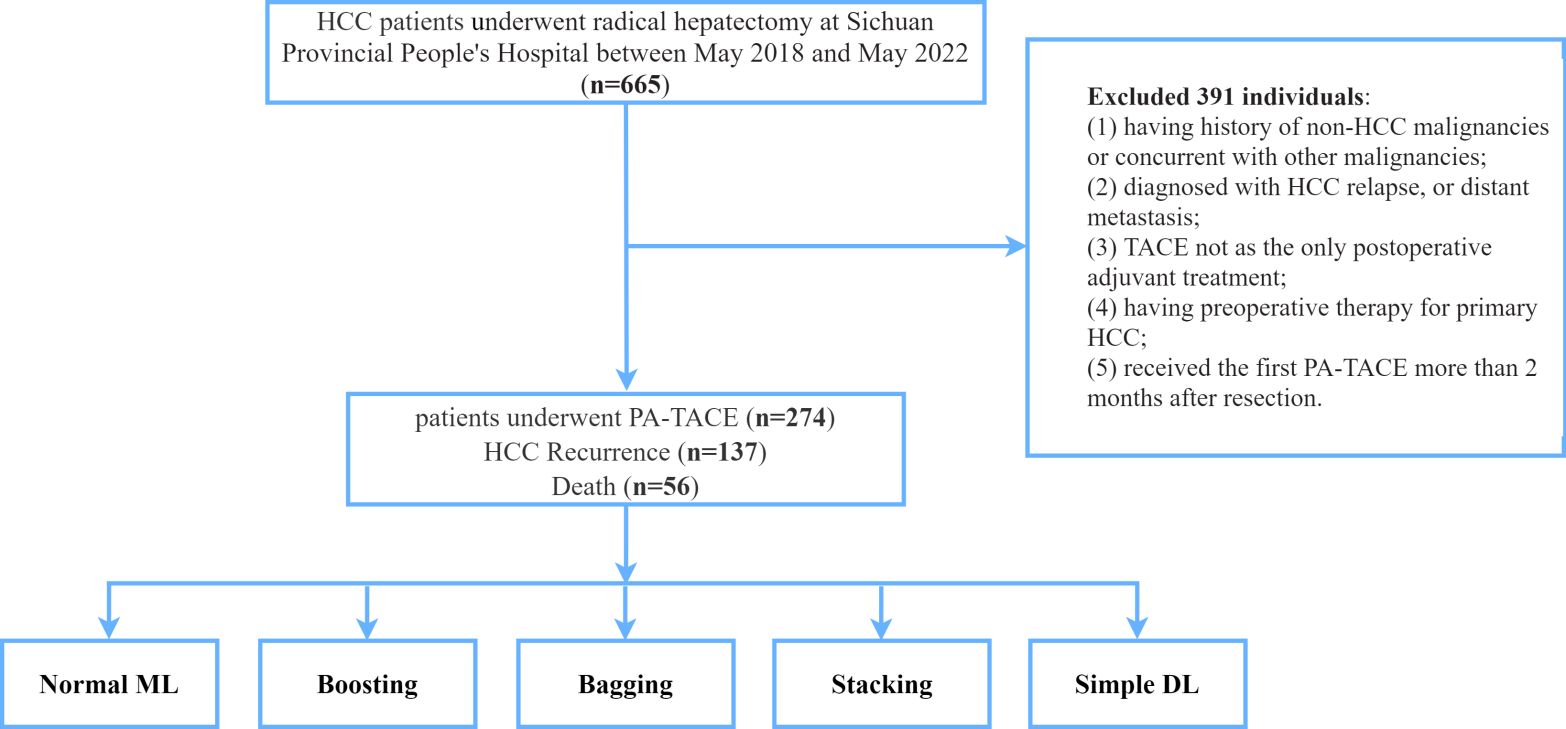
The flowchart of patients enrolled in this study. HCC, hepatocellular carcinoma; PA-TACE, postoperative adjuvant transarterial chemoembolization; ML, machine learning; DL, deep learning.

The database was retrospectively derived from HCC patients who received curative resection and PA-TACE at Sichuan Provincial People’s Hospital between May 2018 and May 2022. The inclusion criteria were included as following: (1) pathological confirmation of HCC; (2) no preoperative therapy for primary HCC; (3) R0 surgical resection of tumor with curative intent; (4) TACE as the only adjuvant treatment; (5) received the first adjuvant TACE within 2 months after resection. Patients who (1) having history of non-HCC malignancies or concurrent with other malignancies; (2) diagnosed with HCC relapse, or distant metastasis; (3) died within 30 days after surgery or lost to follow-up were excluded from this study. The flow chart of the study design can be found in **Figure 1**.

The study was approved by the Human Ethics Committee of Sichuan Academy of Medical Sciences and Sichuan Provincial People’s Hospital, and written informed consent was obtained from all participants. All procedures were performed in accordance with the ethical guidelines of the Helsinki Declaration.

### 2.2 Clinical variables and definitions

The clinicopathological characteristics collected from the database included laboratory tests, tumor characteristics, inflammatory-based prognostic indices, and clinical stages. All laboratory tests were collected within 1 week before the operation, including serum indicators, liver and coagulation functions, and hepatitis B virus markers. The tumor characteristics included differentiation, cirrhosis, the number of tumors, the diameter of the largest nodule, microvascular invasion and so on. Microvascular invasion (MVI) is defined as the presence of HCC microemboli in blood vessels lined by endothelial cells under histological microscope (28). Nowadays, various inflammatory-based prognostic biomarkers are widely used to predict the prognosis of cancer patient. Our study included neutrophil lymphocyte ratio (NLR), platelet lymphocyte ratio (PLR), systemic immune-inflammation index (SII), systemic inflammation response index (SIRI), high sensitivity C-reactive protein (hsCRP)/albumin (ALB), and prognostic nutritional index (PNI). NLR and PLR were calculated as neutrophil/lymphocyte counts and platelet/lymphocyte counts, respectively (29). SII and SIRI were defined as platelet × neutrophil/lymphocyte counts and monocytes × neutrophil/lymphocyte counts, respectively (29, 30). The calculation formula of PNI was as follow: albumin level (g/L) + 5× total lymphocyte count (10^9^/L) (31). The clinical stages included Child-Pugh grade, and Barcelona Clinic Liver Cancer (BCLC) stage.

### 2.3 Follow up

After the surgery, the follow-up was conducted every 3 months in the first year, and then every 6 months thereafter if there was no recurrence or metastasis. The primary outcome was overall survival (OS), which was defined as the time interval from the surgery to death, or the end of the follow-up (July 2022), whichever came first. And the secondary outcome was recurrence-free survival (RFS), which was defined as the time interval from the surgery to death, recurrence, metastasis, or the end of the follow-up (July 2022), whichever came first.

### 2.4 Statistical analysis

Continuous variables were expressed as the medians and interquartile ranges (Q1–Q3), and categorical variables were expressed as frequency (%). Time-dependent ROC curves were used to detect the prognostic performance of the Boosting, Bagging, and Stacking model for OS and RFS, respectively. Survival curves were plotted using the Kaplan-Meier method and the differences were compared by log rank test. The learning rate represents the step size of the model iteration, and the number of estimators means the number of base learners (base models). Two-sided p< 0.05 was considered statistically significant. All statistical analyses were performed using Python version v3.8.10 and GraphPad Prism version 9.2.0.

### 2.5 Model development

#### 2.5.1 Normal machine learning

As a commonly used supervised classification algorithm, KNN (K Nearest Neighbors) has a simple structure and good performance. According to different weight calculation of the neighbor node, there are 2 kinds of model, namely KNN (uniform) and KNN (distance).

If the weight is uniform, the value assigned to a point is calculated according to the simple majority vote of the nearest neighbors. However, in some cases, it is better to weight the neighbors so that the closer neighbors can make more contributions to the fit. Therefore, the second calculation method allocates weights proportional to the reciprocal of the distance from the query point.

#### 2.5.2 Boosting

Boosting is a commonly used ensemble learning strategy. To get better knowledge about how boosting works and why this strategy is useful for clinical data, we used 3 kinds of boosting algorithm in the present study.

XGBoost (Optimized Parallel Tree Boosting) support CART (Classification and Regression Trees) and linear classifier at the same time with high flexibility. The XGBoost with linear classifier can be considered as Logistic regression or linear regression with L1 and L2 regularization. Regularization, which contains the number of leaf node on the tree and L2 regularization of the weight of the leaf node, is be used to balance the model complexity and avoid overfitting. Compared with some deep learning methods, XGBoost has simpler structure and greater interpretability.

Compared with XGBoost, LightGBM gets a breakthrough in memory consumption and calculation speed to some extent. Within histogram algorithm, we change the traversal over samples to traversal on histogram and get performance improvement. At the same time, to speed up further, we present Gradient-based One-Side Sampling (GOSS) to filter out those samples with small gradient. Considering the high calculating speed, the model can be applied to real-time operation (e.g., large-scale medical real-time data analysis).

CatBoost perform roughly between XGBoost and LightGBM, but in view of no parameters adjustment and ordered boosting application to avoid prediction offset, this model wins in simplicity and efficiency.

#### 2.5.3 Bagging

Bagging, including ExtraTrees (Gini, Entr) and RandomForest (Gini, Entr), is also a very commonly used ensemble learning strategy.

As a set of many decision trees, RandomForest perform well on multivariate data due to the composite structure of processing discrete data and continuous data concurrently. There is no need to reduce dimension and choose any other feature selection tools. Also, RandomForest can measure the impact of different attributes, which can help understand multiple indicators of the patient. According to different nodes split type, we construct two kinds of model with Gini coefficient and entropy.

ExtraTrees (Extremely Randomized Trees) enable each base decision tree to use the same original dataset. Overall, ExtraTrees is quite similar to RandomForest except for higher variance and lower bias. Sometimes, ExtraTrees perform better in terms of regularization, so we chose to use this model for comparison in the medical tasks.

#### 2.5.4 Stacking

For models, we consider combining some base learners, including Boosting or Bagging, to perform well. For datasets, we also set a specific training strategy to improve performance of the ensemble model.

**Figure 2:**
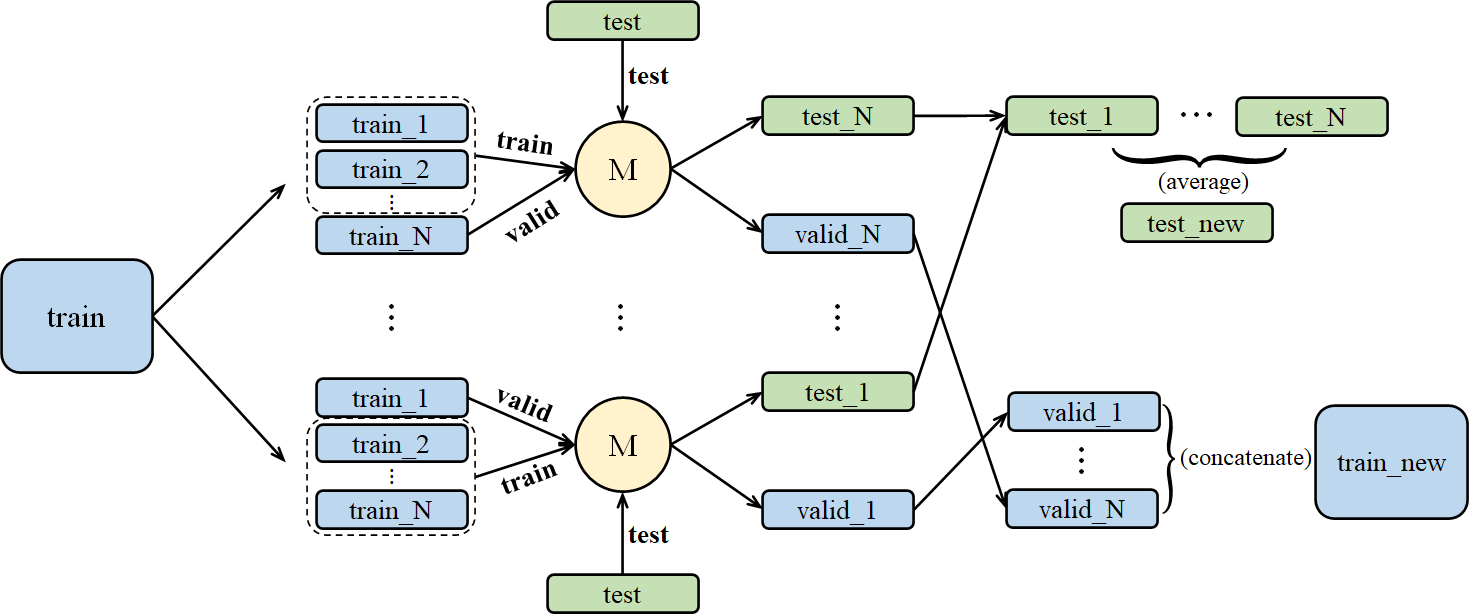
The stacking training process for a single model, including dataset segmentation and result integration.

Stacking aims at building a new model from several base models through feature transformation on training and testing sets. As shown in **Figure 2**, M represents a base model. We divide training set into N pieces and choose 1 piece for validation, while the other N-1 pieces are used to train the base model. After training, we get test and valid results. Then, we take an average of the N test results and get the test set feature transformation. We concatenate N valid results and get the valid set feature transformation.

**Figure 3:**
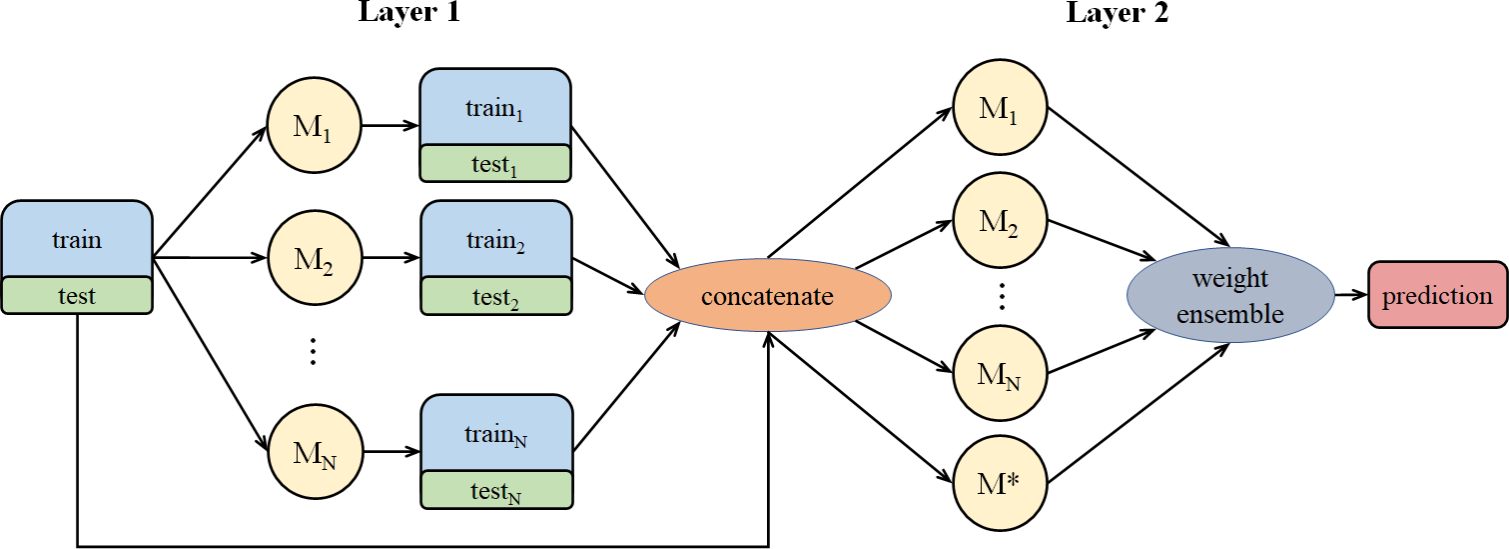
The stacking process of multiple models, including model weight distribution.

For N base models, we proposed the method in **Figure 3**. In **Figure 3**, we concatenate N pairs of train-test and original train-test to get a new dataset and assign it to be the input of the N+1 models. Different from the normal stacking strategy with linear regression(model*) setting on high stacking layer to avoid overfitting, we still set base models on high stacking layer, which can help get better performance. Then we calculate suitable weight combination and get the prediction result from the N+1 models.

#### 2.5.5 Simple deep learning

At first, we did not adopt any deep learning models because of the risk of overfitting and weak interpretability. But AutoGluon offers a different network, which applies different layers for categorical and numerical data.

For multivariate data, individual embedding layers enable the network to learn about each category feature individually before mixed variables to be used as input. In the next step, the embeddings of the category features are concatenated with the number features into a large vector. The vector will be fed into a 3-layer feedforward network and directly connected to the output predictions *via* a linear skip-connection. In AutoGluon, we adopted 2 neural network model based on PyTorch and Fastai v1 respectively.

### 2.6 Model discrimination and calibration

Accuracy, a good and intuitive evaluation index, is commonly used to evaluate the predictive performance of the machine learning models. Generally, higher accuracy means better prediction efficiency. And it can be written as:


$$Accuracy\;=\;\frac{TP\;+\;TN}{TP\;+\;TN\;+\;FP\;+\;FN}$$

(TP: True Positive, TN: True Negative, FP: False Positive, FN: False Negative)

Importance can be used to measure the degree to which different indicators contribute to the model. For example, if we delete one indicator and use the remaining indicators to train a model, the model prediction performance will decline. The degree of the decline, which is the magnitude of the decline in accuracy, is the value of the importance. Therefore, the higher the importance, the more important the indicator will be.

In the present study, different models were constructed using the database and randomly divided into training and validation sets at a ratio of 8:2. We also used K-fold cross-validation to validate the predicative performance of the machine learning model. Because K-fold cross-validation is easy to implement and had skill estimation with lower bias than other methods, it is often used to compare and select models for a given predictive modeling problem. The K-fold cross-validation was performed as follows: Divide the original dataset into K groups (the K value is 10 in our model); Select one group as the test dataset and the remaining groups as the train dataset; Fit a model with the train dataset and test the model on the test dataset; Retain the evaluation score (we use accuracy, precision and recall, and accuracy is the main indicator); Repeat the group selection until each group is tested. In summary, K-fold cross-validation averages K rounds of fitness in the prediction to derive the most accurate estimate of the model prediction performance.

## 3 Results

### 3.1 Patient characteristics and outcome

A total of 274 participants who underwent PA-TACE were recruited in this study, of which 142(51.8%) patients received once PA-TACE treatment. In the prediction model, twenty-eight predictors including clinicopathological characteristics, inflammatory-based prognostic biomarkers and clinical stages were analyzed. The baseline characteristics of all the participants are shown in **Table 1**. At the end of follow-up, 137 (50.0%) patients presented HCC recurrence, and 56 (20.4%) patients died. The median follow-up time of the study was 20 (IQR: 9–30) months.

**Table 1:** Baseline characteristics of the HCC patients.

Characteristics	Overall (n = 274)
Age (years), median (IQR)	56 (48-66)
BMI (kg/m^2^), median (IQR)	22.4 (20.6-23.9)
Sex, n (%)	
Female	34 (12.4%)
Male	239 (87.2%)
Cirrhosis, n (%)	
Yes	197 (71.9%)
No	77 (28.1%)
BCLC stage, n (%)	
0/A	143 (52.2%)
B	96 (35%)
C	35 (12.8%)
Child-Pugh grade, n (%)	
A	204 (74.5%)
B	70 (25.5%)
HBV history, n (%)	
Yes	204 (74.5%)
No	70 (25.5%)
Frequency of PA-TACE, n (%)	
Once	142 (51.8%)
Twice	71 (25.9%)
Third	61 (22.3%)
Microvascular invasion, n (%)	
Positive	104 (38%)
Negative	170 (62%)
Tumor differentiation, n (%)	
Low	65 (23.7%)
Medium‐high	209 (76.3%)
Maximum diameter of tumor (cm), median (IQR)	6 (4-9)
Tumor number, n (%)	
Single	179 (65.3%)
Multiple	95 (34.7%)
Portal vein tumor thrombus, n (%)	
Positive	41 (15%)
Negative	233 (85%)
Albumin (g/L), median (IQR)	38.3 (34.4-40.8)
AFP (ng/ml), median (IQR)	27.92 (4.0-538.7)
hsCRP (mg/L), median (IQR)	4.51 (1.23-15.46)
Total bilirubin (μmol/L), median (IQR)	17.9 (11.9-23.2)
NEUT counts (10^9^/L), median (IQR)	3.44 (2.37-4.59)
LYM counts (10^9^/L), median (IQR)	1.31 (0.97-1.77)
MONO counts (10^9^/L), median (IQR)	0.45 (0.34-0.61)
PT (s), median (IQR)	11.9 (11.1-13.0)
Platelet (10^9^/L), median (IQR)	157 (98-205)
SIRI, median (IQR)	1.13 (0.64-1.94)
SII, median (IQR)	294.1 (131.7-556.8)
PLR, median (IQR)	110.68 (77.17-176.62)
NLR, median (IQR)	2.57 (1.79-4.24)
PNI, median (IQR)	45.28 (40.45-48.73)
hsCRP/ALB, median (IQR)	0.12 (0.03-0.40)

HCC, hepatocellular carcinoma; BMI, body mass index; BCLC, Barcelona Clinic Liver Cancer; AFP, alpha-fetoprotein; ALB, albumin; TACE, transarterial chemoembolization; hsCRP, high sensitivity C-reactive protein; NEUT, Neutrophils; LYM, lymphocyte; MONO, monocyte; PT, prothrombin time; SIRI, systemic inflammation response index; SII, systemic immune-inflammation index; PLR, platelet–lymphocyte ratio; MVI, Microvascular invasion; NLR, neutrophil–lymphocyte ratio; PNI, prognostic nutritional index.

### 3.2 Prediction performance

The machine learning models included in our study were normal ML (KNN), Boosting (XGBoost; CatBoost; LightGBM), Bagging (Extra Trees; Random Forest), Stacking, and simple DL (DeepNN; Fastai). The discriminatory performance of the five models in overall mortality and HCC recurrence were assessed with the accuracy. Among the five models, the ensemble learning strategies, including Boosting, Bagging and Stacking, presented better predictive performance in terms of prognostic risk for HCC patients following PA-TACE (**Tables 2**, **3**). Specially, the accuracy of the Stacking model in predicting overall mortality and HCC recurrence (test-accuracy: 0.8909, valid-accuracy: 0.9318; **Table 2**; test-accuracy: 0.7636, valid-accuracy: 0.8182; **Table 3**) was at the highest level in both training sets and validation sets. Moreover, the fitting time, prediction time and gain of five machine learning models were also compared, indicating that the Stacking model had relatively low time consumption and the best prediction performance (**Tables 2**, **3**).

**Table 2:** Predictive performance of different machine learning models for overall mortality.

	Test-accuracy	Valid-accuracy	Fit time(s)	Pred time(s)	Learning rate	N-estimators	Gain
Normal ML							
KNN (uniform)	0.8182	0.7273	0.0090	0.0385	–	–	-0.0727
KNN (distance)	0.8182	0.7273	0.0094	0.0447	–	–	-0.0727
Boosting							
XGBoost	0.8364	0.8864	0.8183	0.0141	0.1	10000	-0.0545
CatBoost	0.8727	0.9091	1.6993	0.0054	0.05	–	-0.0182
LightGBM	0.8909	0.9091	1.7005	0.3262	0.03	–	0
Bagging							
Extra Trees (Gini)	0.8545	0.8409	1.0892	0.1414	–	300	-0.0364
Extra Trees (Entr)	0.8364	0.8636	1.1028	0.1253	–	300	-0.0545
Random Forest (Gini)	0.8727	0.8409	1.2293	0.1288	–	100	-0.0182
Random Forest (Entr)	0.8364	0.8636	1.0606	0.1164	–	300	-0.0545
Stacking							
Weight Stacking	0.8909	0.9318	0.4780	0.0072	–	–	base
Simple DL							
DeepNN	0.8000	0.7955	1.1492	0.1010	0.0003	–	-0.0909
Fastai	0.7636	0.9091	2.1031	0.2925	0.01	–	-0.1237

ML, Machine Learning; KNN, K Nearest Neighbors; XGBoost, Optimized Parallel Tree Boosting; CatBoost, Gradient Boosting on Decision Trees; LightGBM, Gradient Boosting Framework that Uses Tree Based Learning Algorithms; Extra Trees, Extremely Randomized Trees; Entr, Entropy as Criterion; DL, Deep Learning; DeepNN, Deep Neural Network; Fastai, AutoML Framework.

**Table 3:** Predictive performance of different machine learning models for HCC recurrence.

	Test-accuracy	Valid-accuracy	Fit time(s)	Pred time(s)	Learning rate	N-estimators	Gain
Normal ML							
KNN (uniform)	0.5091	0.6591	0.0091	0.0362	–	–	-0.2545
KNN (distance)	0.5091	0.6364	0.0070	0.0388	–	–	-0.2545
Boosting							
XGBoost	0.6182	0.7273	0.8966	0.0262	0.1	10000	-0.1454
CatBoost	0.7091	0.7727	3.0586	0.0061	0.02	–	-0.0545
LightGBM	0.6727	0.7500	1.1155	0.0847	0.05	–	-0.0909
Bagging							
Extra Trees (Gini)	0.6727	0.7273	1.0993	0.1300	–	300	-0.0909
Extra Trees (Entr)	0.6909	0.7272	1.1894	0.1314	–	300	-0.0727
Random Forest (Gini)	0.7273	0.7727	1.2376	0.1302	–	300	-0.0363
Random Forest (Entr)	0.7455	0.7955	1.1472	0.1354	–	300	-0.0181
Stacking							
Weight Stacking	0.7636	0.8182	0.4634	0.0036	–	–	base
Simple DL							
DeepNN	0.6182	0.7727	2.0419	0.0716	0.0001	–	-0.1454
Fastai	0.7091	0.7500	1.9735	0.2300	0.01	–	-0.0545

HCC, hepatocellular carcinoma; ML, Machine Learning; KNN, K Nearest Neighbors; XGBoost, Optimized Parallel Tree Boosting; CatBoost, Gradient Boosting on Decision Trees; LightGBM, Gradient Boosting Framework that Uses Tree Based Learning Algorithms; Extra Trees, Extremely Randomized Trees; Entr, Entropy as Criterion; DL, Deep Learning; DeepNN, Deep Neural Network; Fastai, AutoML Framework.

**Figure 4:**
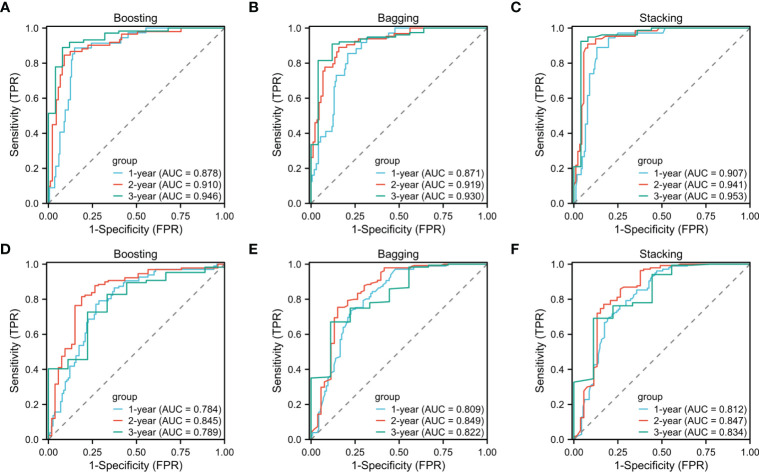
One-, two-, and three-year time-dependent ROC curves for overall survival **(A–C)** and recurrence-free survival **(D–F)** of the Boosting, Bagging and Stacking models.

In time-dependent ROC analysis, the Boosting, Bagging, and Stacking model were found to perform well in predicting OS (1-year: 0.878, 0.871, 0.907; 2-year: 0.910, 0.919, 0.941; 3-year: 0.946, 0.930, 0.953; **Figures 4A–C**) and RFS (1-year: 0.784, 0.809, 0.812; 2-year: 0.845, 0.849, 0.847; 3-year: 0.789, 0.822, 0.834; **Figures 4D–F**) for HCC patients received PA-TACE. Moreover, patients were categorized into low- and high- risk groups based on the median risk score of the Stacking model. The low-risk group had significantly better overall survival and recurrence-free survival than the high-risk group (P<0.001; **Supplemental Figures 1A, B**). Therefore, KM curves indicated good discriminative ability of the Stacking model.

### 3.3 Models and variable importance

We also established the Stacking model to examine the variable importance of recurrence and overall mortality of the HCC patients after PA-TACE. The specific prediction performance of each predictor was measured using importance. The variables with the top 10 importance and P values are also shown in **Table 4**.

**Table 4:** Variable importance of features included in the Stacking model to predict recurrence and overall mortality of the HCC patients after PA-TACE.

No.	Overall Mortality			HCC Recurrence		
	Features	Importance	P value	Features	Importance	P value
1	BCLC Stage	0.029341	0.000158	hsCRP/ALB	0.049635	0.004935
2	AFP	0.021429	0.000053	MVI	0.029197	0.000935
3	ALB	0.014286	0.000076	NLR	0.023358	0.000513
4	Frequency of PA-TACE	0.013106	0.001297	Tumor diameter	0.022628	0.001535
5	hsCRP/ALB	0.011992	0.007032	BCLC Stage	0.019708	0.000674
6	TB	0.011255	0.003995	Frequency of PA-TACE	0.017518	0.000594
7	Cirrhosis	0.010616	0.010858	Tumor differentiation	0.015328	0.002318
8	PT	0.009608	0.003369	Tumor number	0.014599	0.000935
9	PLR	0.008208	0.000121	AFP	0.013869	0.000744
10	Tumor number	0.008126	0.000132	SII	0.012409	0.008770

HCC, hepatocellular carcinoma; BCLC, Barcelona Clinic Liver Cancer; AFP, alpha-fetoprotein; ALB, albumin; TACE, transarterial chemoembolization; hsCRP, high sensitivity C-reactive protein; TB, total bilirubin; PT, prothrombin time; PLR, platelet–lymphocyte ratio; MVI, Microvascular invasion; NLR, neutrophil–lymphocyte ratio; SII, systemic immune-inflammation index.

For overall mortality, the importance of BCLC Stage was 0.029341, substantially higher than scores of other variables, such as AFP (0.021429), ALB (0.014286), and frequency of PA-TACE (0.013106). For HCC recurrence, hsCRP/ALB (0.049635) was the most important variable, followed by MVI (0.029197), NLR (0.023358), and Tumor diameter (0.022628). Furthermore, BCLC Stage, hsCRP/ALB and frequency of PA-TACE were found to be relatively important variables in both overall mortality and recurrence, while MVI contributed more to the recurrence of the patients.

According to different cutoff values, continuous variables were converted into binary variables. Univariate Cox regression analyses were used to determine independent prognostic factors for OS and RFS. The results were generally consistent with the above results obtained from the Stacking model (**Supplemental Table 1**).

## 4 Discussion

To the best of our knowledge, our study was the first to utilize and compare different machine learning algorithms to analyze the RFS and OS outcomes of HCC patients following PA-TACE. Among the five machine learning models, the risk prediction model based on ensemble learning strategies, including Boosting, Bagging, and Stacking algorithms, presented better prediction performance for overall mortality and HCC recurrence. Specially, the fitting time, prediction time and gain of five machine learning models were also compared, indicating that the Stacking model had relatively low time consumption and the best prediction performance. In addition, according to time-dependent ROC analysis, the ensemble learning strategies were found to perform well in predicting both OS and RFS for the patients. In the present study, we also identified the important prognostic factors for postoperative outcomes. We found that BCLC Stage, hsCRP/ALB and frequency of PA-TACE were relatively important variables in both overall mortality and recurrence, while MVI contributed more to the recurrence of the patients.

Nowadays, increasing scoring systems have been developed to evaluate the prognosis of HCC and stratify patients. Most scoring systems have mainly selected significant clinical predictive indices through multivariate analysis, and constructed conventional Cox proportional risk models based on limited risk factors (32–34). However, in clinical studies, various risk factors often have nonlinear effects on recurrence-free survival, especially when they are used in cancer research (35–37). Therefore, the previous traditional models may fail to show the goodness of fitting or make accurate predictions. Machine learning could train algorithms to detect and recognize complex patterns and adapt to more complex nonlinear relationships, thus it might be superior than the traditional models in medical research (25). In our study, machine learning algorithms, including normal machine learning, Boosting, Bagging, Stacking, and simple deep learning were used and compared on RFS and OS outcomes of HCC patients received PA-TACE. The results showed that the Stacking algorithm, an ensemble learning strategy, presented relatively low time consumption, good discriminative ability, and the best prediction performance for the clinical outcomes. Therefore, this ensemble learning model, based on routine peripheral blood cell measurements and clinical characteristics, provides an easily accessible, effective, and intelligent approach for predicting OS and RFS in HCC patients received PA-TACE. Admittedly, the clinical practicability of this model needs further investigation.

In the present study, we also focused on the adaptability of different ensemble learning strategies to clinical data, so that other researchers could better apply specific ensemble learning models to specific clinical data through our comparative experiments. Considering the limited data size and complex data combinations of multiple attributes and dimensions used to predict the prognosis of HCC patients receiving PA-TACE, we therefore developed ensemble learning models including Boosting, Bagging, and Stacking algorithms, rather than sophisticated deep learning strategies. These models do not have excessive overfitting, nor do they have a large number of hyperparameter to learn, which ensures a certain degree of generalization of the model. Moreover, K-fold cross-validation could be used to average K rounds of fitness in the prediction to derive the most accurate estimate of the model prediction performance. In conclusion, we believe that the ensemble learning model developed in our study could also be used to predict the prognosis in subsequent small sample clinical data with multiple attributes and dimensions.

The majority of liver cancers occur in cirrhotic livers with chronic inflammation, which creates a pro-inflammatory environment that promotes tumor formation and development (38–40). The importance of host inflammatory responses indicates the role of inflammatory indices in predicting clinical outcomes of the cancer patients (41). Therefore, apart from the laboratory tests and tumor characteristics, several inflammatory-based prognostic indices (NLR, PLR, SII, SIRI, hsCRP/ALB and PNI) were also included in this study. According to the feature importance analysis based on the Stacking model, interesting outcomes were obtained using these variables. Specifically, BCLC Stage, hsCRP/ALB and frequency of PA-TACE were relatively important variables in predicting both overall mortality and recurrence, while MVI contributed more to the recurrence of the patients. These findings are supported by the following studies.

Firstly, treatment allocation and prognostic stratification based on BCLC staging system, which is closely related to the prognosis of HCC, are currently the most widely used guidelines in clinical practice (42). For HCC patients, those in the early stages of BCLC could obtain good survival and low recurrence prognosis after curative resection (42, 43). In the present study, the predictive performance of the BCLC stage ranked first and fifth in overall mortality and recurrence of patients undergoing PA-TACE, which is in line with the results of previous studies.

Secondly, preoperative hsCRP is considered to be the most sensitive protein synthesized by the liver to detect systemic inflammation and could reflect the burden or development of HCC tumor cells (44, 45). In addition, preoperative albumin is an effective factor to reflect the nutritional status and liver function of the patients, and it is also a decisive factor of tumor cell immune response (46, 47). Studies have also shown that hsCRP/ALB and hsCRP/LYM have a powerful prognostic value for recurrence outcomes in HCC (48, 49). Therefore, for patients receiving PA-TACE, hsCRP/ALB might be a better prognostic indicator than other features, especially in predicting HCC recurrence.

Thirdly, the basic principle of postoperative TACE is to remove tumor cells that may have been shed from the resected tumor mass during hepatectomy and to eliminate small intrahepatic metastases that may not have been detected before or during the operation (50). Therefore, several studies have proved that postoperative adjuvant TACE could improve the prognosis of HCC patients (12, 13, 50). Specifically, the frequency of PA-TACE is also associated with a reduced HCC recurrence rate, improving the long-term prognosis of patients (12). Furthermore, tumor dissemination and spread through microvessels might be one of the reasons for advanced tumor, tumor progression, and poor prognosis (51). Consistent with our results, MVI was also one of the unique parameters in many prognostic models for surgically resected HCC, including Early Recurrence After Surgery for Liver Tumor (ERASL), Singapore Liver Cancer Recurrence (SLICER) and Surgery-Specific Cancer of the Liver Italian Program (SS-CLIP) models (52–54).

This study also has several limitations. First, our model is primarily based on patients from one Chinese center with a limited sample size. It is necessary to validate our findings in further international, multicenter, large-scale studies. Second, the follow-up period is relatively short, long-term outcomes from prospective studies are critical to further extend the performance of our model.

## 5 Conclusion

In conclusion, we have utilized and compared models based on different machine learning algorithms and found that the ensemble learning models could better predict the risk of mortality and recurrence in individual HCC patients following PA-TACE. Specially, the Stacking algorithm presents relatively low time consumption, good discriminative ability, and the best predictive performance for clinical outcomes. Machine learning models could also help clinicians identify the important prognostic factors that are clinically useful in individualized patient monitoring and management.

## Data availability statement

The raw data supporting the conclusions of this article will be made available by the authors, without undue reservation.

## Ethics statement

The studies involving human participants were reviewed and approved by theHuman Ethics Committee of Sichuan Academy of Medical Sciences and Sichuan Provincial People’s Hospital. All procedures were performed in accordance with the ethical guidelines of the Helsinki Declaration. The participants provided their written informed consent to participate in this study.

## Author contributions

YL, ZW and YP were responsible for study conception and design, data acquisition, data analysis and drafting and revision of the manuscript. XH, JS were responsible for study conception and design, data analysis and drafting and revision of the manuscript. ZD, CL, YQ, YY and YS were responsible for data analysis and drafting and revision of the manuscript. All authors contributed to the article and approved the submitted version.
