# Supplementary material for: Development of ensemble learning models for prognosis of hepatocellular carcinoma patients underwent postoperative adjuvant transarterial chemoembolization
Source: Front Oncol. 2023 May 26;13:1169102. doi: 10.3389/fonc.2023.1169102 (PMC10254793; doi:10.3389/fonc.2023.1169102)
Supplement: Supplementary file 1 [file DataSheet_1.pdf]

# *Supplemental material*

## Development of ensemble learning models for prognosis of hepatocellular carcinoma patients underwent postoperative adjuvant transarterial chemoembolization

Yuxin Liang [1,3], Zirui Wang [2], Yujiao Peng [1], Zonglin Dai [1,3], Chunyou, Lai [1,3], Yuqin Qiu [1], Yutong Yao [1,3], Ying Shi [1,3], Jin Shang [1,3]*, Xiaolun Huang [1,3]*

***Correspondence:** Xiaolun Huang Email: huangxiaolun@med.uestc.edu.cn

**Supplemental Figure 1** Kaplan-Meier curves of the Stacking model for overall survival (A) and recurrence-free survival (B).

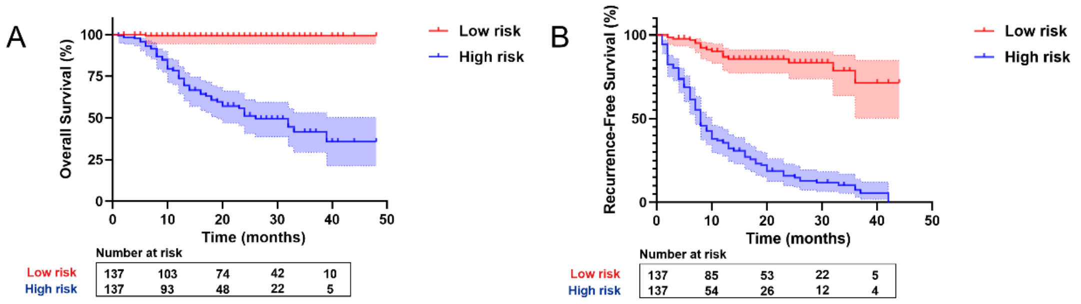


**Supplemental Table 1** Univariate Cox regression analyses of the associations between the prognostic factors and overall survival and recurrence-free survival of the HCC patients.

| | **Overall survival** | | **Recurrence-free survival** | |
|---|---|---|---|---|
| | **Univariate Analysis** | | **Univariate Analysis** | |
| **Characteristics** | **HR (95% CI)** | **P value** | **HR (95% CI)** | **P value** |
| BCLC Stage (0-A vs. B-C) | 2.952 (1.676-5.200) | **<0.001** | 1.992 (1.412-2.809) | **<0.001** |
| hsCRP/ALB (≤0.36 vs. >0.36) | 5.163 (2.979-8.946) | **<0.001** | 2.229 (1.575-3.154) | **<0.001** |

| | | | | |
|---|---|---|---|---|
| Tumor number<br>(Single vs. Multiple) | 1.977<br>(1.169-3.345) | **0.011** | 1.746<br>(1.240 -2.460) | **0.001** |
| Microvascular invasion<br>(No vs. Yes) | 3.466<br>(2.004-5.995) | **<0.001** | 2.539<br>(1.808-3.567) | **<0.001** |
| PLR<br>(≤110.14 vs. >110.14) | 2.486<br>(1.392-4.440) | **0.002** | 1.462<br>(1.041-2.053) | **0.029** |
| NLR<br>(≤3 vs. >3) | 1.972<br>(1.160-3.354) | **0.012** | 1.540<br>(1.099-2.159) | **0.012** |
| AFP, ng/mL<br>(≤233.75 vs. >233.75) | 3.278<br>(1.924-5.584) | **<0.001** | 1.898<br>(1.350-2.688) | **<0.001** |
| Tumor diameter, cm<br>(≤6.3 vs. >6.3) | 3.509<br>(1.960-6.281) | **<0.001** | 1.962<br>(1.398-2.753) | **<0.001** |
| ALB, g/L<br>(≤40 vs. >40) | 0.397<br>(0.188-0.838) | **0.015** | 0.752<br>(0.508-1.113) | 0.154 |
| Frequency of PA-TACE<br>(Once vs. More than once) | 0.497<br>(0.290-0.852) | **0.011** | 0.524<br>(0.361-0.761) | **<0.001** |

**Abbreviations**: HCC, hepatocellular carcinoma; BCLC, Barcelona Clinic Liver Cancer; hsCRP, high sensitivity C-reactive protein; PLR, platelet–lymphocyte ratio; NLR, neutrophil–lymphocyte ratio; ALB, albumin; AFP, alpha-fetoprotein; TACE, transarterial chemoembolization.

**Note**: Bold values means the P value is significant.
